# Regulation of Hippo-YAP1/TAZ pathway in metabolic dysfunction-associated steatotic liver disease

**DOI:** 10.3389/fphar.2025.1505117

**Published:** 2025-01-23

**Authors:** Wei Xuan, Dandan Song, Jianghua Hou, Xiuping Meng

**Affiliations:** ^1^ Department of Hepatopancreaticobiliary Surgery, China-Japan Union Hospital, Jilin University, Changchun, China; ^2^ Department of Clinical Laboratory, Second Hospital of Jilin University, Changchun, China; ^3^ Department of Endodontics, Hospital of Stomatology, Jilin University, Changchun, China

**Keywords:** Hippo-YAP1/TAZ pathway, metabolism, senescence, ferroptosis, inflammation, fibrosis, MASLD

## Abstract

Metabolic dysfunction-associated steatotic liver disease (MASLD) has become the most prevalent chronic liver disease worldwide, but effective treatments are still lacking. Metabolic disorders such as iron overload, glycolysis, insulin resistance, lipid dysregulation, and glutaminolysis are found to induce liver senescence and ferroptosis, which are hot topics in the research of MASLD. Recent studies have shown that Hippo–YAP1/TAZ pathway is involved in the regulations of metabolism disorders, senescence, ferroptosis, inflammation, and fibrosis in MASLD, but their complex connections and contrast roles are also reported. In addition, therapeutics based on the Hippo–YAP1/TAZ pathway hold promising for MASLD treatment. In this review, we highlight the regulation and molecular mechanism of the Hippo–YAP1/TAZ pathway in MASLD and summarize potential therapeutic strategies for MASLD by regulating Hippo–YAP1/TAZ pathway.

## 1 Introduction

Metabolic dysfunction-associated steatotic liver disease (MASLD) is a metabolic disorder that can develop into metabolic dysfunction-associated steatohepatitis (MASH) which can lead to liver fibrosis, cirrhosis, and even cancer (Lazarus et al., 2022). High-fat obesity is the most prevalent cause of MASLD, affecting 20%–30% of the global population. Metabolic disorders such as iron overload, glycolysis, insulin resistance, lipid dysregulation, and glutaminolysis are the basis of MASLD (Chen et al., 2020). However, effective treatments for MASLD are still lacking, and underlying molecular mechanisms remain unclear.

The Hippo pathway is first discovered to regulate cell density and organ size through its downstream effectors, Yes-associated protein 1 (YAP1) and transcriptional coactivator with PDZ-binding motif (TAZ) (Russell and Camargo, 2022). Hippo-YAP1/TAZ pathway can regulate the inflammation of macrophages or the fibrogenic program of hepatocytes by binding to the TEA domain (TEAD) family (Mia and Manvendra, 2022). In addition, Hippo–YAP1/TAZ pathway has been recently found to regulate metabolic disorders, which induce senescence and ferroptosis, which are hot toptics in the research of MASLD (Nguyen-Lefebvre et al., 2021). Cellular senescence, a hallmark of aging, is characterized by a stable arrest of the cell cycle and a secretion of pro-inflammatory proteins known as senescence-associated secretory phenotypes (SASP), which promote liver fibrosis (López-Otín et al., 2013). Ferroptosis, a new manner of cell death characterized by iron-dependent phospholipid peroxidation, is also found to regulate cell death and inflammation in the pathological process of MASLD (Ogrodnik et al., 2019; Minamino et al., 2009). However, the research of Hippo–YAP1/TAZ pathway in MASLD are still in its infancy and their contrast roles on senescence, ferroptosis, inflammation, and fibrosis are also found.

In this review, we highlight the regulation and mechanism of the Hippo-YAP1/TAZ pathway in metabolism disorders, cellular senescence, ferroptosis, inflammation, and fibrosis on the progression of MASLD, which may offer new insights for developing effective treatment strategies for MASLD.

## 2 Hippo-YAP1/TAZ signaling pathway

Hippo signaling is first identified to regulate organ growth (Moya and Halder, 2019; Ma et al., 2015). Hippo signaling senses extracellular mechanical stimuli and triggers a kinase cascade, the mammalian sterile 20-like kinase 1/2 (MST1/2), and large tumor suppressor 1 and 2 (LATS1/2). MST1/2 phosphorylates its adaptor protein salvador 1 (SAV1) to facilitates its interaction and phosphorylation with LATS1/2. MST1/2 also phosphorylates MOB kinase activator 1A/B (MOB1A/B), enabling MOB1A/B to bind the auto-inhibitory region of LATS1/2 and promote full LATS1/2 activation (Moya and Halder, 2019; Ma et al., 2015; Hansen et al., 2015). The phosphorylation of LATS1/2 can further phosphorylate YAP1 and TAZ, leading to their retention in the cytoplasm and subsequent proteasomal degradation (Hansen et al., 2015). Inhibition of YAP1 and TAZ phosphorylation facilitate them to bind with TEAD as transcriptional coactivators, thus regulating various cellular behaviors (d’Angelo et al., 2019).

Mitogen-Activated Protein Kinase 4 (MAP4K) family, 4 Fat molecules (FAT1-4), and thousand and one kinases (TAOK1/2/3), have been found to phosphorylate LATS1/2 directly, or through activation of the MST1/2 kinases, respectively (Yu and Guan, 2013; Angus et al., 2012; Xiao et al., 2011; Boggiano et al., 2011; Poon et al., 2011; Meng et al., 2015; Zheng et al., 2015). In contrast, striatin-interacting phosphatase and kinase (STRIPAK) complex was found to inactivate MST1/2 (Zheng Y. et al., 2017; Bae et al., 2017), while G protein-coupled receptors (GPCRs) family was found to inhibit LATS1/2 activity, thus inhibiting the phosphorylation of YAP1/TAZ and promoting their nuclear translocation to activate transcription factors (Hansen et al., 2015; Feng et al., 2014; Yu et al., 2014; Cai et al., 2018; Zhao et al., 2012). Notably, many other post-translational modifications such as O-GlcNAcylation are also reported to affect YAP1 phosphorylation by interrupting the LATS1/2 interaction (Zhang et al., 2017a).

## 3 Pathological regulations of metabolic disorders on MASLD

Metabolic disorders including iron, glucose, and lipid dysregulations are the basis of MASLD. For example, the imbalance of iron homeostasis has been shown to correlate with obesity and the development of MASLD (Folgueras et al., 2018). Dietary iron intake correlated with the increased prevalence of MASLD in a dose-responsive manner (Alferink et al., 2019). Pathological iron overload can catalyze the generation of reactive oxygen species (ROS) through the Fenton reaction, contributing to chronic liver disease (Nakamura et al., 2019; Lunova et al., 2014). In addition, insulin resistance, enhanced glycolysis activity, increased lactic acid, and abnormal lipid accumulation are also found to increase the inflammatory cytokines, and cause liver damage and fibrosis in MASLD or MASH (Ye et al., 2016; Reyes-Gordillo et al., 2017).

Metabolic disorders have been found to induce senescence, which is a permanent cessation of the cell proliferation cycle, induced by internal or external factors (Kuilman et al., 2010). For example, palmitic acid (PA) can promote iron overload and induce senescence of hepatocytes (Qi et al., 2021). Senescent cells exhibit aberrant oxidative stress, excessive production of pro-inflammatory cytokines known as SASP, and mitochondrial dysfunction, which contribute to the progression of MASLD (Dabravolski et al., 2021). Inhibition of hepatocyte senescence could improve the pathological process of MASLD (Ogrodnik et al., 2017).

Metabolic disorders such as iron and lipid homeostasis impairments have been found to promote MASLD development by inducing ferroptosis, which is a new manner of cell death characterized by iron-dependent phospholipid peroxidation (Kuilman et al., 2010). Iron-export protein ferroportin 1 is downregulated, and iron-import proteins DMT1, transferrin receptor 1 (TfR1), and ferritin are upregulated in MASLD (Philpott, 2020; Mancias et al., 2014; Liu et al., 2022; Mitsuyoshi et al., 2009). Iron overload has been shown to exacerbate liver injury in a dose-responsive manner in a phase 2 trial of MASLD (Beaton et al., 2013). Similarly, a high-iron diet for 8 weeks can induce mouse liver ferroptosis and damage (Yu et al., 2020). Ferroptosis initiates cell death and inflammation at the onset of MASH by directly increasing the expression of prostaglandin endoperoxide synthase 2, which accelerates the metabolism of arachidonic acid and promotes inflammation (Tsurusaki et al., 2019). Inhibition of ferroptosis alleviates hepatic inflammatory responses and steatosis (Qi et al., 2020). Ferritinophagy is involved in regulating iron metabolism and ferroptosis (Lunova et al., 2014). Nuclear receptor coactivator 4 (NCOA4) is required for the delivery of ferritin to the lysosome via autophagosomes. Deferoxamine (DFO), an iron-chelating agent, inhibits lipid accumulation in the liver of MASH mice induced by ferroptosis inducer RSL3 (Shao and Chung, 2024).

## 4 Regulation of Hippo-YAP1/TAZ pathway in MASLD

Metabolites are also found to regulate the Hippo-YAP1/TAZ pathway. For example, elevated glucose levels enhance YAP1 activity by promoting O-GlcNAc modification or inhibiting adenosine monophosphate-activated kinase (AMPK) (Zhang et al., 2017b; Peng et al., 2017). In contrast, glucose starvation increased Hippo pathway phosphorylation and YAP1/TAZ cytoplasmic localization via activation of AMPK (Wang et al., 2015a). G-protein-coupled receptor (GPCR) signaling has also been discovered to control Hippo and YAP1/TAZ signaling (Yu et al., 2012). Glucagon or phospholipids such as lysophosphatidic acid can increase LATS1/2 activity and YAP1 phosphorylation by activating GPCR (Yu et al., 2013). Cholesterol-induced activation of TAZ is linked to lipid dysregulation and MASLD pathogenesis in mice (Wang et al., 2020). Mechanically, high-level cholesterol activates and stabilizes TAZ by triggering the inositol trisphosphate receptor (IP3R)-calcium-RhoA pathway in AML12 cells (Wang et al., 2020).

The Hippo-YAP1/TAZ pathway is also found to regulate metabolic disorders. Moreover, the Hippo-YAP1/TAZ pathway is involved in the regulation of senescence and ferroptosis, inflammation, and fibrosis caused by metabolic disorders in the MASLD (Sztolsztener et al., 2020).

### 4.1 Regulation of the Hippo-YAP1/TAZ pathway in metabolic disorders in MASLD

Hippo signaling has been reported to regulate metabolic abnormalities in MASLD (Minamino et al., 2009). For example, YAP1 can modulate iron concentration in MASLD patients by affecting TfR1 and ferritinophagy (Protchenko et al., 2021; Zhu et al., 2021a). NCOA4 is one of the specific mediators of ferritinophagy that degrade ferritin, which is critical for iron homeostasis (Philpott, 2020; Mancias et al., 2014). In addition, YAP1 is also found to suppress gluconeogenesis, a normal process in which the liver produces glucose in the fasted state (Hu et al., 2017). Mechanically, YAP1 represses the transcription of peroxisome proliferator-activated receptor gamma co-activator-1 alpha (Pgc1α), which plays an important role in gluconeogenesis (Hu et al., 2017). YAP1 can also drive glycolysis partially through the regulation of the glucose transporter glucose transporter 3 (GLUT3), which promotes glucose uptake (He et al., 2021). Moreover, YAP1 was found to induce glycolysis by up-regulating long noncoding RNA BCAR4, which was reported to increase the expression of hexokinase 2 (Zheng X. et al., 2017). Key metabolic enzyme AMPK regulates glucose metabolism by directly phosphorylating YAP1 (Wang et al., 2015b). Hippo-YAP1/TAZ signaling was also found to promote glucose metabolism by up-regulating insulin receptor substrate 2 (Irs2) and protein kinase B (Akt) (Wang C. et al., 2016). YAP1 can also activate Foxm1, which is a crucial regulator of insulin resistance and lipid metabolism and contributes to the progression of MASLD (Jeong et al., 2018). Moreover, Hippo signaling is involved in lipid metabolism by regulating the expression of MST1, which inhibits cholesterol accumulation by reducing constitutive sterol regulatory-element binding protein (SREBP) activity (Aylon et al., 2016; Geng et al., 2016). Consistently, SREBF2 promotes cholesterol and fatty acid synthesis by driving the expressions of HMGCR and FASN (Geng et al., 2016; Romani et al., 2019). Long non-coding RNA LncARSR is found to promote the accumulation of fat in the liver by targeting YAP1 in MASLD by potentiating IRS2/AKT activity. In addition, YAP1 is found to increase glutamine levels in zebrafish liver by directly promoting the expression of glula and glulb (Cox et al., 2016). Furthermore, YAP1 promotes glutaminolysis and myofibroblastic features in the hematopoietic stem cells (HSCs) of patients and mice with acute or chronic fibrosis (Du et al., 2018).

### 4.2 Regulation of the Hippo-YAP1/TAZ pathway in senescence and ferroptosis in MASLD

Hippo-YAP1/TAZ pathway is involved in the regulation of liver senescence. The YAP1 level is reduced and the iron level is increased in LO2 cells treated with PA. Over-expression of YAP1 can mitigate iron overload and alleviate hepatocyte senescence by inhibiting ferritinophagy (Qi et al., 2021). It is favored that over-expression of NCOA4 largely aggravates lipid droplet deposition and cellular senescence in PA‐treated LO2 cells (Qi et al., 2021). LncRNA MAYA is also increased in PA‐treated LO2 cells and high-fat diet (HFD)‐induced hepatic steatosis in MASLD mice. Suppression of MAYA alleviates iron overload and cellular senescence by up-regulating YAP1 expression (Yuan et al., 2021). Selectively inhibition of YAP1 in HSCs induces senescence, but decreases liver injury and fibrosis by promoting ferroptosis resistance (Du et al., 2023).

The Hippo-YAP1/TAZ pathway can affect many essential ferroptosis pathways, such as the cystine/glutathione (GSH)/glutathione peroxidase 4 (GPX4) axis, the guanosine triphosphate cyclohydrolase 1 (GCH1)/tetrahydrobiopterin (BH4)/dihydrofolate reductase (DHFR) axis, and the ferroptosis suppressor protein 1 (FSP1)/coenzyme Q (CoQ) axis (Zheng and Conrad, 2020). Moreover, YAP1 increased the iron concentration in hepatocellular carcinoma cells (HCC) through transcriptional elevation of TfR1 via its O-GlcNAcylation (Zhu et al., 2021b). Downregulation of YAP1 partially reversed Fibroblast growth factor 21 (FGF21)-mediated ferroptosis of HCC (Xia et al., 2024). TAZ is also reported to mediate ferroptosis by indirectly regulating the expression of the ROS-generating nicotinamide adenine dinucleotide phosphate oxidass (NOX) in ovarian cancer cells (Yang et al., 2020). The p53/YAP1 axis is essential for lipid peroxidation and ferroptosis induced by cytoglobin, a heme-binding protein, that is critical for maintaining redox homeostasis within cells (Ye et al., 2021). However, YAP1/TAZ is also found to inhibit lipid peroxidation and ferroptosis by up-regulating the expressions of solute carrier family 7 member 11 (SLC7A11) and lipoxygenase 3 (Chalasani et al., 2012; Qin et al., 2021). Consistently, YAP1 knockdown aggravated liver injury and inflammation, as well as accelerated hepatocyte ferroptosis induced by cecal ligation and puncture (Wang et al., 2022). Increase in YAP1 nuclear translocation can inhibit ferroptosis of HSCs in carbon tetrachloride (CCl4)-induced mouse liver fibrosis model (Zhu et al., 2024).

Evidence regarding the role of YAP1/TAZ in senescence and ferroptosis within the liver remains controversial. Nevertheless, the regulatory mechanisms of YAP1/TAZ in senescence and ferroptosis have not been thoroughly in the context of the MASLD.

### 4.3 Regulation of the Hippo-YAP1/TAZ pathway in inflammation and fibrosis of MASLD

YAP1 activation was found to suppress the infiltration of macrophages and neutrophils and reduce the activation of HSCs and liver fibrosis (Liu et al., 2019). YAP1 was increased in macrophages derived from the livers of human cirrhotic patients or CCl4-induced mice (Feng et al., 2024). YAP1 acts as a coactivator for β-catenin, which regulates its target gene X-box binding protein 1, resulting in decreased NOD-like receptor protein 3/caspase-1 activity in macrophages isolated from mouse livers (Wu et al., 2021). It was favored that MST1 can promote reactive oxygen species (ROS)-induced pyroptosis accompanied by decreased YAP1 level in mouse pancreatic cancer model (Cui et al., 2019). MST1/2 depletion in liver macrophages enhanced liver inflammation and fibrosis accompanied by increased YAP1 level in MASLD (Zhang J. et al., 2024). Pharmacological inhibition of YAP1 in KCs has been shown to attenuate the expression of inflammatory cytokines and liver damage in NASH mice (Song et al., 2020). Mechanically, YAP and TAZ are found to promote the production of inflammatory cytokines in MASH mice by enhancing the secretion of Indian hedgehogs (Song et al., 2020; Wang X. et al., 2016). YAP1/TAZ is also reported to promote inflammation in metabolically stressed hepatocytes by up-regulating the expression of cysteine-rich angiogenic inducer 61 (CYR61) in mice (Mooring et al., 2020). The transcription factor Nrf2 mitigates YAP1-mediated inflammation by reducing reactive oxygen species (ROS) and MST1/2 phosphorylation (Wang et al., 2019). Notably, chronic inflammation also prolongs YAP1/TAZ activation in murine MASLD models and liver biopsies from patients with MASLD (Hyun et al., 2021). Pro-inflammatory cytokines including TNFα can directly modulate hepatocyte YAP1 activity in a dose-dependent manner (Zhao et al., 2020), suggesting that the interplay between YAP1/TAZ and inflammatory signaling is complex.

In addition, YAP1/TAZ can directly activate a fibrogenic program of HSCs via regulation of proliferation (Swiderska-Syn et al., 2016). YAP1-positive ductular liver cells were correlated with pro-fibrogenic factors (Tgfβ1, Ctgf, and phosphor-SMAD2) in MASH (Machado et al., 2015). Integrin β1 is required for activating pro-fibrotic hepatocytes by promoting YAP1 expression and its nuclear localization (Martin et al., 2016). The pharmacological blockade of YAP1 with verteporfin prevented collagen formation in cultured mouse HSCs (Martin et al., 2016). Omega-3 polyunsaturated fatty acids have been reported to inhibit the proliferation and activation of HSCs by degradating YAP1/TAZ (Zhang et al., 2016). It is favored that YAP1 promotes the proliferation of cardiac fibroblasts and their transdifferentiation to myofibroblasts in a murine dilated cardiomyopathy model (Jin et al., 2019). Notably, hepatocyte proliferation is generally beneficial for repopulating tissues and YAP1/TAZ is critical for liver regeneration by regulating neurofibromatosis-2 (NF2), which is a negative regulator of liver regeneration (Benhamouche et al., 2010). Inhibiting YAP1/TAZ rescues hepatocyte- and cholangiocyte-overgrowth phenotypes of Nf2-knockout mice (Benhamouche et al., 2010). Pharmacological inhibition or genetic deletion of MST1/2 enhances liver regeneration (Lu et al., 2018). Loss of integrin-linked kinase increased YAP1 expression and liver regeneration following partial hepatectomy (Apte et al., 2009). Precise consequences of YAP1/TAZ activity depend on the overall health of the liver micro-environment (Miyamura et al., 2017).

## 5 Treatment targeting Hippo-YAP1/TAZ pathway in MASLD

By focusing on the YAP1/TAZ pathway, new pharmacological treatments or MASLD might be found. Si-Ni-San is one of the representative formulas for treating patients with MASLD and is used to reduce lipid droplet deposition and YAP1 expression in the liver in mice (Wang et al., 2021). Lian-Mei-Yin, a traditional Chinese medicine formula, has been used for treating liver disorders by inhibiting YAP1-mediated Foxm1 activation, which is crucial for the occurrence and development of MASLD in zebrafish and mice (Zhang P. et al., 2024). Curcumol is an ingredient extracted from the volatile oil of a traditional Chinese herb zedoary turmeric and inhibits hepatocyte senescence by targeting YAP1/NCOA4 in MASLD (Sorrentino et al., 2014). Statins have been widely used to reduce cholesterol levels in patients with hypercholesterolemia and have recently been identified to inhibit YAP1/TAZ through Rho-mediated modulation of actin cytoskeleton, indicating that statins might be used for treating MASLD (Wang et al., 2015c). In addition, hepatocyte-specific N-acetylgalactosamine-conjugated siRNA targeting TAZ is sufficient to ameliorate HFD-induced liver fibrosis in mice (Liu-Chittenden et al., 2012). Furthermore, verteporfin, MYF-01–37, and XMU-MP-1 have been discovered to interact with YAP1 with TEAD, suggested that they might be used to treat MASLD in the future (Martin et al., 2016; Liu-Chittenden et al., 2012; Kurppa et al., 2020; Fan et al., 2016).

## 6 Conclusion and perspectives

**FIGURE 1:**
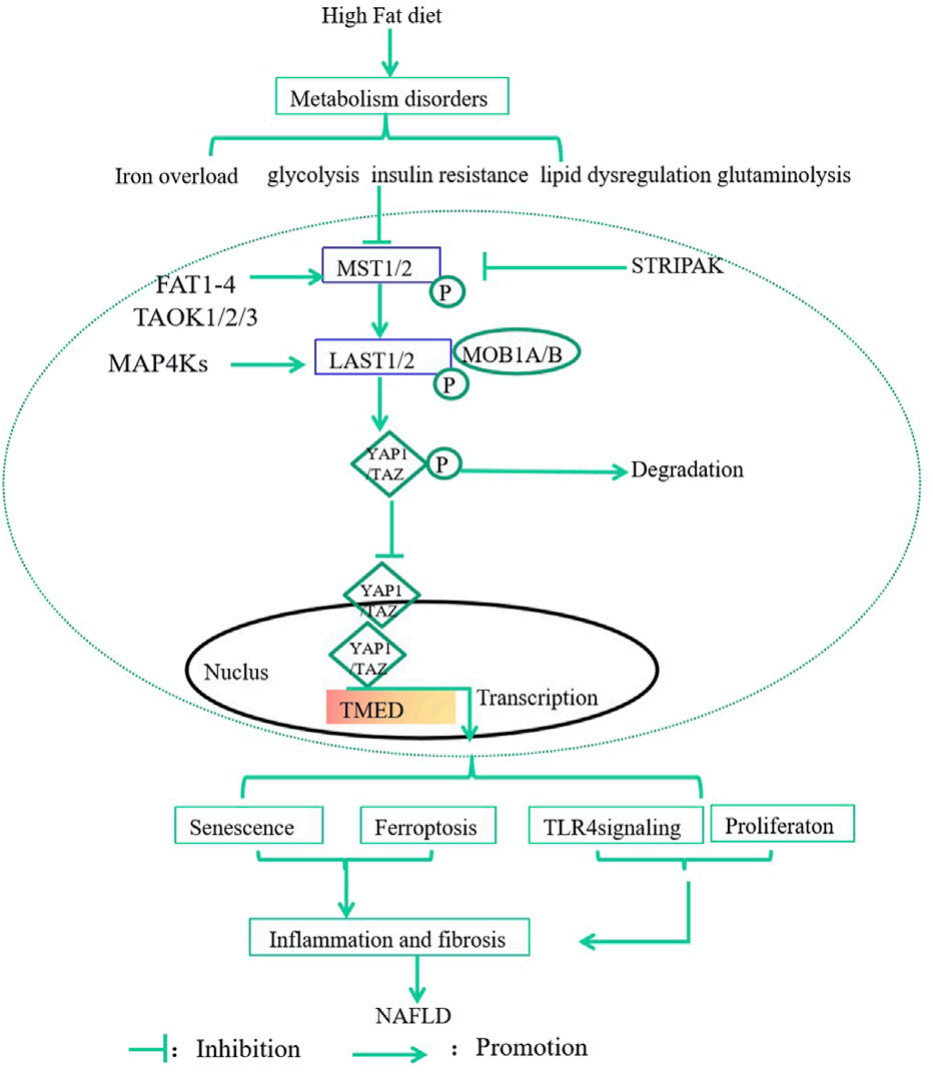
The effects and mechanisms of Hippo-YAP1/TAZ Pathway in MASLD. Metabolic disorders such as iron overload, glycolysis, insulin resistance, lipid dysregulation, and glutaminolysis can induce senescence, ferroptosis, inflammation, and fibrosis to promote the development of MASLD via regulation of Hippo-YAP1/TAZ Pathway. MST1/2, 20-like kinase 1/2; LATS1/2, large tumor suppressor 1 and 2; P, phosphorylation; MAP4K, Mitogen-Activated Protein Kinase 4; MOB1A/B, MOB kinase activator 1A/B; TAOK, thousand and one kinases; GPCRs, G protein-coupled receptors; STRIPAK, striatin-interacting phosphatase and kinase; FAT; Fat molecule.

In this review, we highlight the regulations and mechanisms of Hippo- YAP1/TAZ signaling in MASLD by focusing on metabolism, senescence, ferroptosis, inflammation, and fibrosis. Metabolic disorders such as iron overload, glycolysis, insulin resistance, lipid dysregulation, and glutaminolysis can induce senescence, ferroptosis, inflammation, and fibrosis to promote the development of MASLD via regulation of Hippo-YAP1/TAZ pathway (Figure 1). The drugs targeting Hippo-YAP1/TAZ signaling might be used to treat MASLD in the future. However, the interplay between YAP1/TAZ and inflammation or fibrosis is complex, precise consequences of YAP1/TAZ activity depend on the overall health of the liver micro-environment, which need further investigation.
